# Widespread Polycistronic Transcripts in Fungi Revealed by Single-Molecule mRNA Sequencing

**DOI:** 10.1371/journal.pone.0132628

**Published:** 2015-07-15

**Authors:** Sean P. Gordon, Elizabeth Tseng, Asaf Salamov, Jiwei Zhang, Xiandong Meng, Zhiying Zhao, Dongwan Kang, Jason Underwood, Igor V. Grigoriev, Melania Figueroa, Jonathan S. Schilling, Feng Chen, Zhong Wang

**Affiliations:** 1 Department of Energy Joint Genome Institute, Walnut Creek, California, United States of America; 2 Pacific Biosciences, Menlo Park, California, United States of America; 3 Department of Bioproducts & Biosystems Engineering, University of Minnesota, Saint Paul, Minnesota, United States of America; 4 Department of Plant Pathology, University of Minnesota, Saint Paul, Minnesota, United States of America; 5 School of Natural Sciences, University of California at Merced, Merced, California, United States of America; Albert Einsten College of Medicine, UNITED STATES

## Abstract

Genes in prokaryotic genomes are often arranged into clusters and co-transcribed into polycistronic RNAs. Isolated examples of polycistronic RNAs were also reported in some higher eukaryotes but their presence was generally considered rare. Here we developed a long-read sequencing strategy to identify polycistronic transcripts in several mushroom forming fungal species including *Plicaturopsis crispa*, *Phanerochaete chrysosporium*, *Trametes versicolor*, and *Gloeophyllum trabeum*. We found genome-wide prevalence of polycistronic transcription in these Agaricomycetes, involving up to 8% of the transcribed genes. Unlike polycistronic mRNAs in prokaryotes, these co-transcribed genes are also independently transcribed. We show that polycistronic transcription may interfere with expression of the downstream tandem gene. Further comparative genomic analysis indicates that polycistronic transcription is conserved among a wide range of mushroom forming fungi. In summary, our study revealed, for the first time, the genome prevalence of polycistronic transcription in a phylogenetic range of higher fungi. Furthermore, we systematically show that our long-read sequencing approach and combined bioinformatics pipeline is a generic powerful tool for precise characterization of complex transcriptomes that enables identification of mRNA isoforms not recovered via short-read assembly.

## Introduction

Advances in sequencing technologies have led to the discovery of an enormous variety of RNA species within cells, including both coding and non-coding RNAs[1,2], splicing isoforms[3], alternatively polyadenylated isoforms[4,5], and gene-fusion transcripts[6–8]. High-throughput short-read sequencing of transcriptomes (RNA-Seq) has enabled a precise quantification of gene expression levels and the identification of new exons and splice junctions[9,10]. However, as short-reads are much shorter than the length of most transcripts, assembly of these short-reads is necessary to infer the full cornucopia of transcript diversity[11]. For organisms that lack a reference genome, *de novo* transcriptome assembly from short-reads is often the only available choice. However, transcript assembly has many informatics challenges as it involves piecing together large volumes of short-reads to reconstruct individual transcript isoforms[11]. The largest challenges of short-read assembly include resolving hundreds of distinct isoforms derived from the same loci, and overlapping transcripts on the same strand for transcripts that span different loci[8,12,13]. Reduced sensitivity of short-read assembly to identify multiple isoforms from the same locus and long multi-locus transcripts clouds our ability to accurately define transcriptional units.

With a mean read length of >7 kb, the Pacific Biosciences (PacBio) single-molecule sequencing platform provides a direct and unbiased observation of full-length transcripts and their diversity. The throughput of the technology has dramatically increased, making genome-wide transcriptome studies possible for eukaryotes[14–16]. To overcome the low single-pass sequencing accuracy of the platform, recent studies either used circular consensus (CCS) reads[15] or 2^nd^ generation short-reads to correct errors in PacBio long-reads[16,17]. The CCS correction strategy excludes long transcripts (>3kb) and thus has limited ability to analyze long RNAs, while the short-read correction strategy requires additional sequencing efforts and the short-read sequencing may have biased coverage over transcripts with extreme GC-content. Thus, additional approaches are needed to fully utilize PacBio long-reads for comprehensive transcriptomics studies.

In this study we developed a transcriptome sequencing and analysis strategy called ToFU (Transcript isOforms: Full-length and Unassembled) that requires only PacBio reads for generating a *de novo* transcriptome, eliminating the need for short-read assembly or reference genomes. We chose to test ToFU on four wood-degrading basidiomycete fungal transcriptomes, *Plicaturopsis crispa*, *Phanerochaete chrysosporium*, *Trametes versicolor* and *Gloeophyllum trabeum*[18] as these fungi possess genomic characteristics that are ideal to examine the effectiveness of our approach. First, these basidiomycete fungi have genes with higher intron numbers and more prevalent alternative splicing than ascomycetes and thus rich RNA isoform diversity[19]. Second, despite exhibiting complex alternative splicing, intron-rich basidiomycetes have smaller numbers of expressed loci than many higher eukaryotes, which makes them an ideal candidate for testing ToFU. Finally, the biochemical and physiological adaptations of these fungi to decompose wood represent a mechanism with great biotechnological potential in engineering plant biomass deconstruction and advancing synthetic biology. Our knowledge related to RNA transcript isoform diversity in intron-rich fungi is limited as they are under-represented in transcriptome studies, and little is known about isoform diversity of mRNAs encoding the enzymes that govern wood-degrading processes. With these in mind, we first deeply sequenced the transcriptome of the white-rot basidiomycete *P*. *crispa* with both short- and long-read technology to benchmark our approach. Subsequently, we generated additional long-read transcript sequences for three additional species (*P*. *chrysosporium*, *T*. *versicolor* and *G*. *trabeum*) representing different orders within the Basidiomycota and showed the existence of widespread long polycistronic mRNAs in these fungi.

## Results

### A single-molecule, long-read strategy to identify full-length isoforms

**Fig 1:**
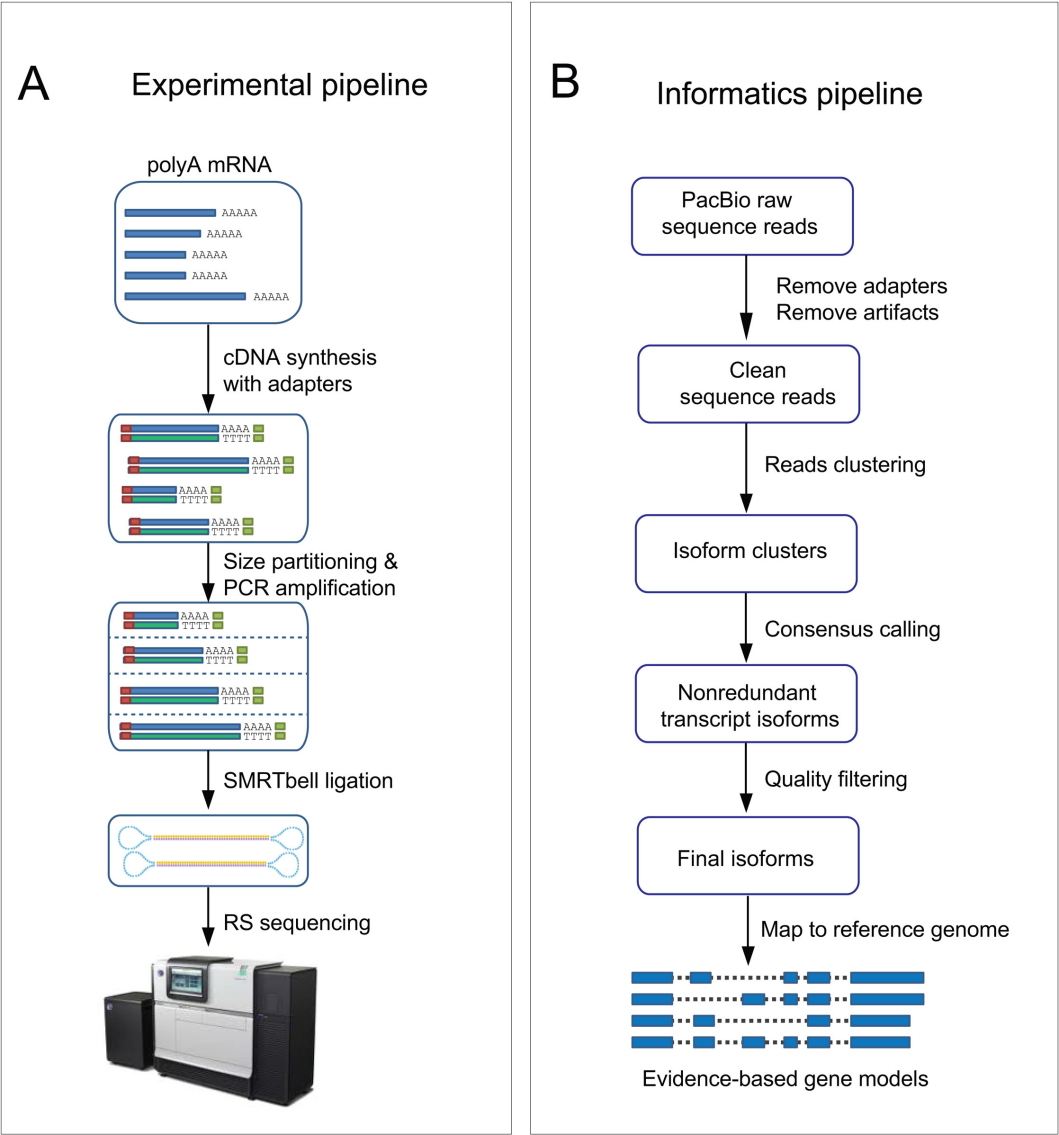
An overview of the experimental (a) and informatics(b) components in the ToFU pipeline to generate transcript isoforms.

The goal of ToFU was to bypass complicated experimental and informatic procedures of short-read assembly and instead leverage the longest reads from the PacBio platform to yield high-confidence transcript isoforms independent of a reference genome and therefore making the approach applicable to any organism (Fig 1 and Methods). To increase the representation of different mRNA populations in *P*. *crispa*, multiple cDNA libraries, including size selected (1–2 kb, 2–3 kb, and 3–6 kb) and non-size selected libraries, were generated and sequenced for each of two growth conditions. After sequencing, we identified putative full-length cDNA reads from 5 million raw reads by the presence of both 5’ cDNA primers and polyA signals preceding the 3’ primers, yielding 2.1 million full-length sequences. Reads derived from the same isoforms were then clustered to generate initial consensus sequences, and further polished with the aid of non-full-length reads to generate 176,903 high-quality consensus sequences. After merging redundant sequences we obtained 22,956 distinct isoforms representing 9,073 transcribed loci (Table A in S1 File). In the following sections, we denote this final set of isoforms as the ToFU transcript set. For performance comparison and validation purposes, we also independently generated standard short read RNA-Seq data (300 million paired-end 100bp reads) on the Illumina HiSeq platform from the same RNA samples.

### ToFU transcripts are long and accurate

**Fig 2:**
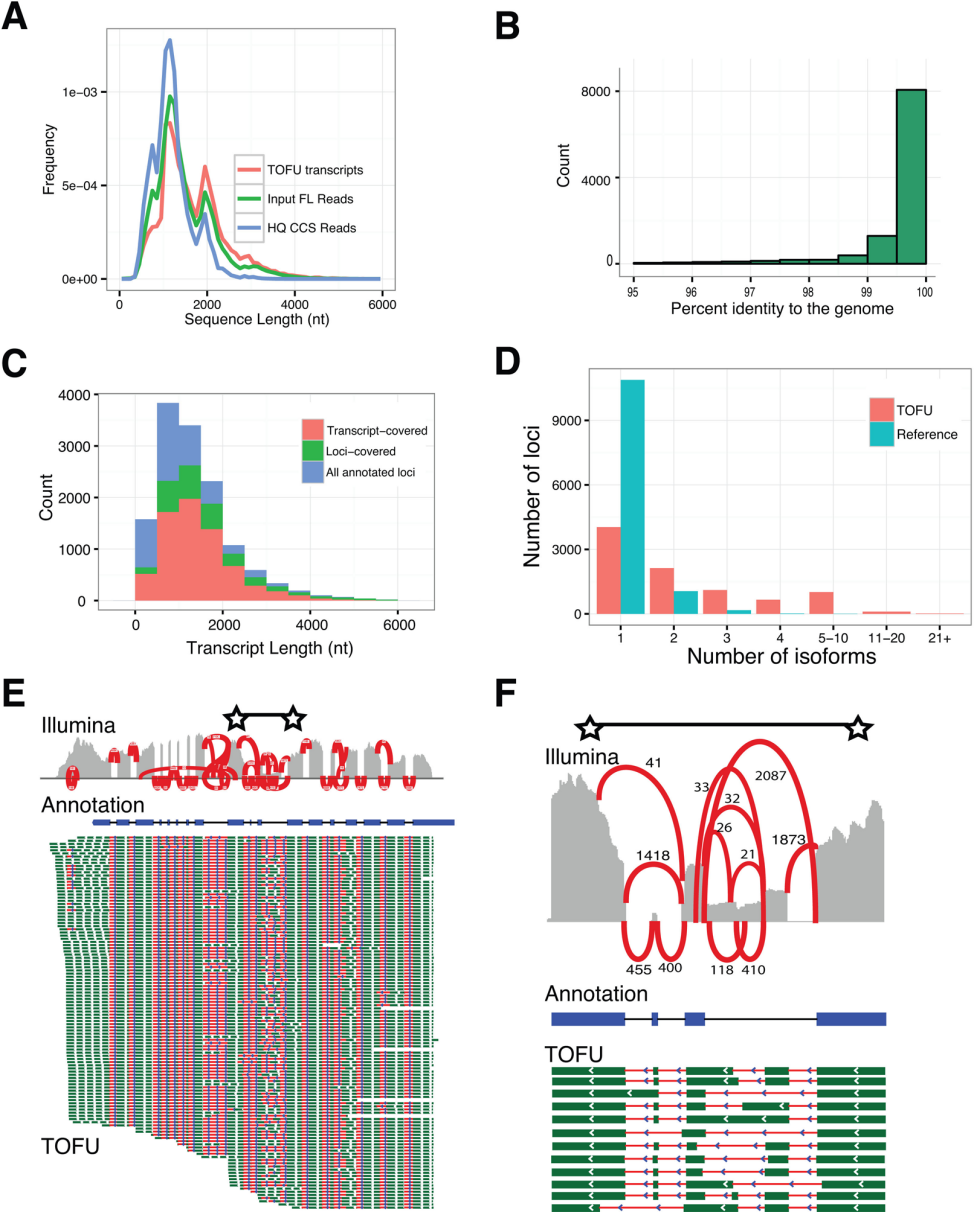
Long, high-quality, consensus sequences accurately benchmark transcript diversity. **a**, Length distributions of full-length (FL) input reads, high-quality CCS reads, and ToFU transcript sequences. **b**, Histogram of percent nucleotide identity of ToFU transcript sequences aligned to the reference genome. **c**, Accumulative histogram of number of reference annotations that have a ToFU transcript that completely covers each annotated junction (transcript-covered) or only partially covers the annotated gene (loci-covered). Reference annotations that were not assayed (blue stack) are also shown. **d**, Distribution of distinct isoforms per loci for the reference annotation and ToFU transcript set. **e**. Illumina short-read coverage (grey) and junction support (red lines, associated numbers indicate Illumina reads that support each splice junction) aligned along the reference annotated transcript (blue) for a glycosyl hydrolase gene with 120 distinct PacBio isoforms aligned below (splice junctions are shown in red and exon sequences are shown in green). **f**, An enlarged view of the region between two starts in **2e**.

The ToFU transcripts (Fig 2A) have an average length of 1,657 nt, with the longest being 5,589 nt. The apparent length limitation to 6kb is most likely a combined result of ineffective size selection and the limitation of the sequencing chemistry (P4-C2, Methods) used in this study; it is also unclear how many transcripts > 6 kb are present in *P*. *crispa*. The length of the final transcripts closely follows the distribution of the input full-length reads (*Input FL Reads* in Fig 2A) since no assembly is involved. ToFU transcripts include a large number of isoforms greater than 3 kb that are not accessible by simply using CCS reads (*HQ CCS Reads*, Fig 2A)[15].

Despite the ~15% error rate in the input reads[17], our analyses indicate that ToFU transcripts are highly accurate. Although our pipeline did not require a sequenced genome, we used the annotated draft genome sequence of *P*. *crispa* from JGI MycoCosm portal[20] (http://jgi.doe.gov/Plicaturopsis) to independently estimate transcript accuracy. When aligned to the genome sequence using GMAP[21] and allowed alignment gaps, 99.79% (37,930,451/38,011,774) of the bases are concordant with the reference base (Fig 2B). The estimated errors for substitution, insertion, and deletion are 0.06%, 0.04% and 0.12% respectively. These percentages are likely over-estimated since they do not account for errors in draft reference genome, polymorphisms, or post-transcriptional RNA-editing. In addition, based on existing reference-based gene annotations[20] the ToFU transcripts fully span most of the genes with detected expression (Fig 2C, transcript-covered versus loci-covered) and on average they have longer untranslated regions (UTRs) (Fig A in S2 File).

### ToFU reveals extensive alternative splicing (AS) and alternative poly-adenylation

Fungal species were previously thought to have much lower rates of alternative splicing than plants and animals. Recent estimates based on EST and RNA-Seq data suggest that on average approximately 7.3% of genes in non-Saccharomycotina fungi undergo AS, with *Cryptococcus neoformans* being an extreme case with up to 20% of genes involved in AS[19]. By contrast, 42% of genes in Arabidopsis and 95% in humans are alternatively spliced[22,23]. Among 9,073 transcribed loci in *P*. *cripsa*, 56% (5,038 / 9,073) have two or more and 32% (2,908) have three or more distinct isoforms that derived from either alternative splicing, alternative poly-adenylation, or alternative transcription start sites (Fig 2D). In total, 25.2% of all transcribed loci are alternatively spliced and 28.7% loci have alternative poly-adenylation sites. This estimation of splicing rate is likely underestimated, as rare isoforms may skip detection, and we only sampled two conditions. These findings suggest that basidiomycete fungi may have a much higher transcriptional diversity than previously reported.

Wood-decaying fungi produce a wide range of enzymes to break down plant cell walls including a large and diverse family of glycosyl hydrolases (GHs). Despite their importance, little is known about GH transcript isoform diversity at individual genes, which may affect the efficiency at which these enzymes are made both in their native host and bioengineered systems. Interestingly, among 151 loci that have 10 or more isoforms, 8 are associated with GH activity. One of these GH loci produces 120 distinct isoforms, with additional support from short-read validation of individual splice junctions (Fig 2E and 2F).

### A quality evaluation of short-read assemblers

**Fig 3:**
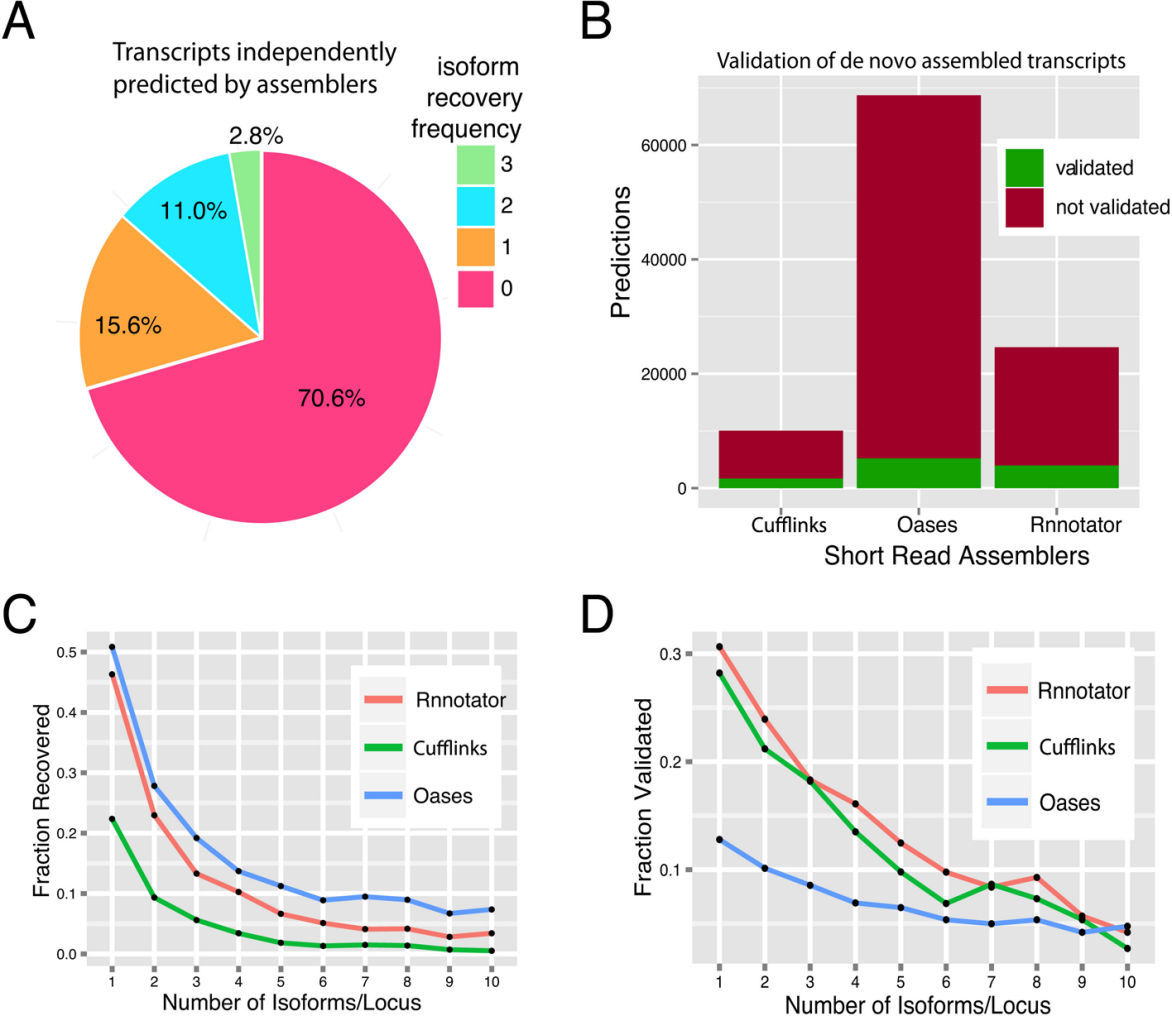
Evaluating short-read transcript reconstruction against ToFU transcripts. **a**, Percentage of ToFU transcripts recovered by three different short-read assembly methods. The isoform frequency shows whether a ToFU transcript is recovered by exactly 0, 1, 2, or all 3 of the assemblers. **b**, Number of assembled transcripts validated by ToFU transcripts. A transcript is validated as an exact match of a ToFU transcript if it shares exactly the same number of exons and donor-acceptor sites. **c**, Fraction of ToFU transcripts recovered (sensitivity) by each short-read assembler as a function of isoform complexity. **d**, Fraction of assembled transcripts validated (specificity) by ToFU as a function of isoform complexity. Isoform complexity is determined by the number of ToFU isoforms at each locus.

Extensive alternative splicing in the *P*. *crispa* transcriptome makes it a good candidate to assess the quality of algorithms for transcriptome reconstruction from short-reads. In order to quantify the ability of existing short-read transcript reconstruction methods to capture isoform level resolution we used the ToFU transcript set as a reference. There are quite a few published tools for *de novo* transcriptome assembly. As in this work the goal is to compare short and long read sequencing for transcriptome assembly, we selected three representative assemblers to represent both genome-based (Cufflinks[24]) and *de novo* (Rnnotator[25] and Oases[26]) reconstruction strategies. All assemblies were generated from the above 300 million 100-bp paired end short-read dataset. The performance of each assembler was evaluated by its ability to recover ToFU transcripts (sensitivity) and the number of predictions validated by ToFU (specificity) (Fig 3). For a fair comparison, we only considered loci that were detected by both short-reads and ToFU transcripts, and we evaluated the reconstructed transcripts only based on their exon structures (splicing junctions).

Overall, a single assembler was only able to reconstruct a small percentage of 22,956 ToFU isoforms, and only 2.8% of isoforms by all three methods (Fig 3A). 70% of ToFU transcripts were not fully reconstructed by any of the three assemblers. Among the three short-read assemblers, Oases had the largest number of transcripts and the highest prediction sensitivity, but it also had the most predictions not validated by ToFU and thus the least specificity. Cufflinks seemed to be the most conservative assembler, predicting only a small number of transcripts compared with the other two. Rnnotator showed a balance between sensitivity and specificity, with only one-third as many transcripts predicted as Oases with similar sensitivity (Tables B and C in S1 File, Fig 3B). Importantly, both the sensitivity (Fig 3C) and specificity (Fig 3D) of all the above assemblers dropped sharply as isoform complexity increased.

The above analyses highlight the limitations of current state-of-the-art short-read assembly methods for isoform discovery, and suggest that long-read RNA sequencing is essential for accurate isoform resolution, especially for genes with many isoforms.

### Long-read sequencing reveals widespread polycistronic mRNAs in *P*. *crispa*

**Fig 4:**
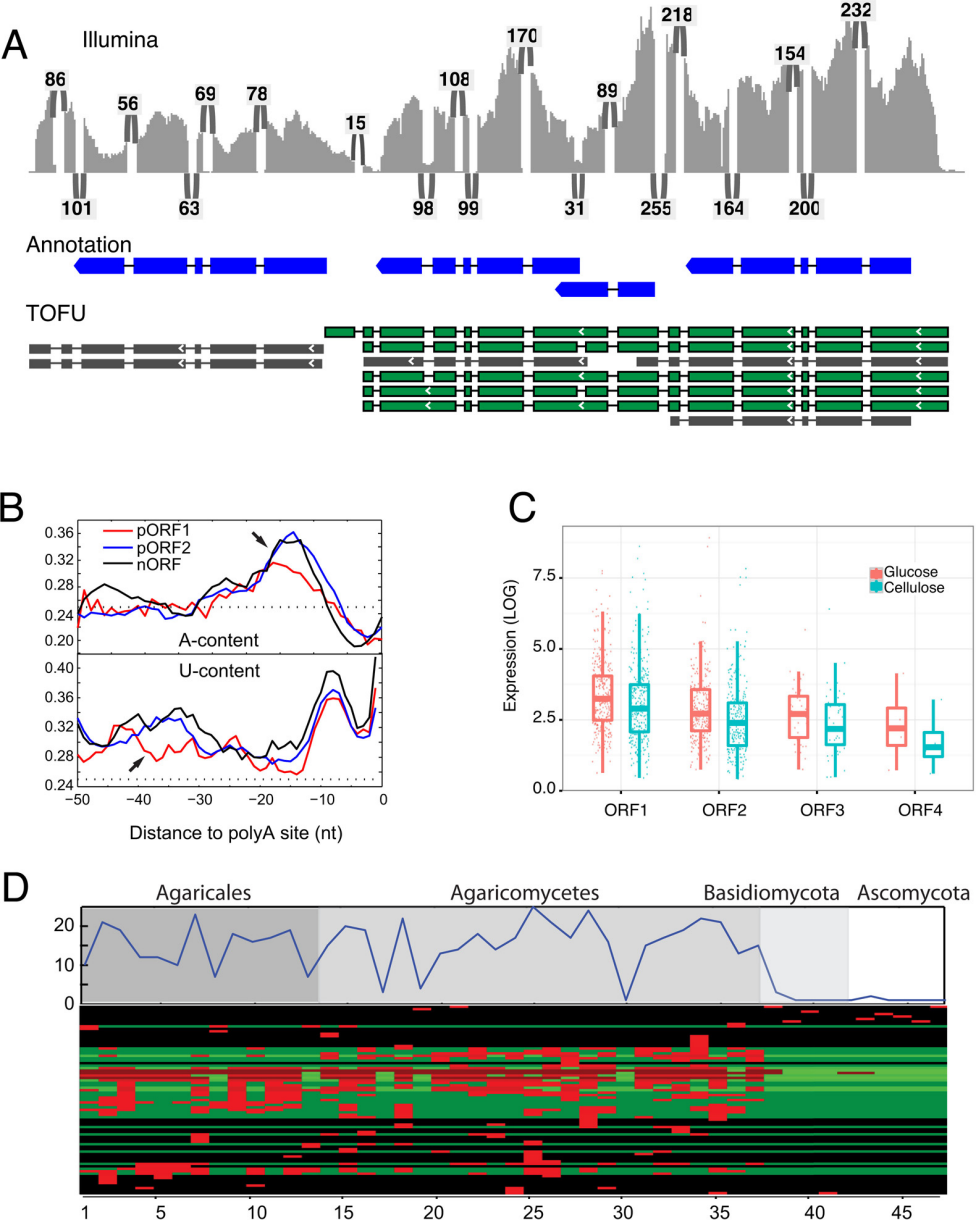
The genome-wide presence of polycistronic mRNAs. **a**, Short-reads (Illumina) aligned to a cluster of tandem reference genes (Annotation, 3 tandem genes on the first row). The numbers of supporting short-reads for each junction are indicated. Polycistronic transcripts (TOFU) are shown in green and non-polycistronic transcripts in gray. **b**, A comparison of transcription termination signals. The sequence composition profiles (upper panel for A-content and lower panel for U-content) before the polyadenylation sites for different classes of ORFs. pORF1 is the upstream ORF and pORF2 is downstream ORF, while nORF stands for non-polycistronic mRNAs. The y-axis are the frequencies of a specific nucleotide averaged for 200 randomly sampled polycistronic mRNA or non-polycistronic controls, dotted lines are the expected frequencies (0.25) if all four bases are equally likely. Arrows denote NUE (upper panel) and FUE (lower panel), respectively. For this figure, only polycistronic transcripts with exactly two ORFs are plotted. Genome-wide analysis base composition of termination signals for all transcribed loci is shown in Fig B in S2 File **c**, The independent expression levels of ORFs within polycistronic RNAs. ORF numbers indicate their order in the transcript (5’- to 3’). **d**, Polycistronic transcripts are likely a unique feature to Agaricomycetes. The top plot shows the total number of adjacent ORF pairs within polycistronic transcripts from *P*. *crispa* that have conserved gene configuration in related species. The numbers on x-axis are species with increasing evolutionary distance. The bottom heatmap shows the conservation for each individual pair of ORFs. Red indicates the presence of a homologous gene pair in the species.

Detailed analysis of the opening reading frames (ORFs) of the *P*. *crispa* ToFU transcript set revealed three-hundred and fourteen loci with one or more readthrough transcripts that overlapped two or more annotated reference genes. They collectively involve 717 of the 9,073 transcribed loci (7.9%). 234 multi-gene loci were associated with a readthrough transcript that completely contained two to four annotated independent ORFs on the same strand (an example is shown in Fig 4A). Multiple stop codons are present in all reading frames between these ORFs, excluding the possibility that the transcripts are large single ORFs that are misannotated.

Unlike the small regulatory upstream ORFs found in yeast and many other organisms[9,27], the average size of the upstream ORFs is comparable to the downstream ones (256 vs 277 amino acids) with an average inter-ORF distance of 364 nt. We therefore subsequently refer to the loci generating polycistronic transcripts as polycistronic transcription units (PTUs). The majority of loci with identified PTUs had polycistronic transcripts in two experimental conditions derived from independent libraries. PTU transcripts were further validated by mapping to the reference genome, continuous coverage support from short-read mapping to the PTU transcripts (minimum of 10 short-read mapped bases), and junction support from short-read mapping to the reference genome. Together the evidence suggests that these transcripts are accurate polycistronic mRNAs as they are confirmed by multiple independent sources (PacBio sequencing, Illumina short reads, and reference gene annotation).

PTUs are a common feature of the prokaryotes, but are relatively rare in eukaryotes except for transpliced transcripts in protists and nematodes[28]. To our knowledge this is the first report of extensive PTUs in higher fungi. To rule out the possibility that these transcripts are experimental or informatics artifacts, we carried out independent validation experiments by RT-PCR followed by additional sequencing of amplicons (Methods and Table D in S1 File). In support of the high fidelity of long-read sequencing strategy, 8 out of 10 randomly selected polycistronic transcripts were successfully validated by PCR and sequencing, while the remaining 2 were inconclusive due to technical PCR problems.

In humans, *Arabidopsis thaliana* and the filamentous ascomycete *Aspergillus oryzae*, signals for transcription termination include an A-rich near upstream element (NUE), and a U-rich far upstream element (FUE)[4,29,30]. The PTUs could result from transcriptional readthrough due to weak termination signals[4]. To address this possibility, we compared the sequence composition before the poly-adenylation sites of the upstream and the downstream ORFs. First, we did a genome-wide analysis of sequences surrounding poly-adenylation sites to confirm the presence of NUE and FUE elements in the basidiomycete, *P*. *crispa* (Fig B in S2 File). We then compared the termination signals of ORF1 in polycistronic transcripts (Fig 4B, pORF1) against its downstream ORF (Fig 4B, pORF2) and non-polycistronic transcripts (Fig 4B, nORF). Consistent with the weak transcription termination hypothesis, ORF1 is lacking both the U-rich FUE and A-rich NUE.

### Genes within polycistronic transcripts are also independently transcribed

Unlike in prokaryotes where polycistronic genes are transcribed into a single transcript without independent transcription, the polycistronic genes in *P*. *crispa* are also independently transcribed (Fig 4A). However, expression of downstream genes is consistently lower than their upstream counterparts within the same polycistronic transcript and this trend was consistent in independent experimental conditions (Fig 4C). Thus genes associated with readthrough transcripts frequently formed 2 to 4 successive tiers of decreasing gene expression. Polycistronic readthrough RNAs associated with this biased expression are different than previously identified regulatory RNAs such as lncRNAs[1] that fall largely outside genic regions or transcripts that are found in antisense orientation relative to genes due to convergent readthrough transcription[31,32]. This raises the possibility that the expression of downstream genes is repressed by the upstream readthrough transcription either through transcriptional interference (TI) or nucleosome positioning in which intergenic transcription alters the organization of nucleosomes at promoters thus influencing their activity[33,34].

### Polycistronic RNAs are conserved among a wide range of Agaricomycetes

To investigate the evolutionary origin of these genome-wide PTUs from *P*. *crispa*, we used the pairs of adjacent ORFs within these transcripts as queries to search 47 sequenced fungal genomes (Table E in S1 File) for conserved gene configurations. These fungal species include 13 species from the same subclass as P. crispa (Agaricomycetidae), 24 from the same class (Agaricomycotina), and 33 from the same phylum (Basidiomycota). In addition, there are 4 species from the phylum Ascomycota. Since there are no available long read sequences from these species, we reasoned that conserved gene configuration would be indicative of possible readthrough transcription in other species. These conservation analyses indicate that a subset of the gene pairs have conserved configuration in multiple species (Fig 4D), but this conservation declines outside of the Agaricomycete class. This suggests that the gene pairs in P. crispa PTUs may also produce polycistronic transcripts in other Agaricomycetes.

To validate the notion that other Agaricomycetes produce PTUs, we generated additional PacBio transcriptome data from three species from different orders than *P*. *crispa*, but within Agaricomycetes class. These fungi represent three independent orders (Polyporales, Gloeophyllales, Amylocorticiales), and include two additional white rot fungi *Phanerochaete chrysosporium*, and *Trametes versicolor*, as well as one brown rot fungus *Gloeophyllum trabeum* (Methods). Even without deep sequencing, we identified at least a hundred putative polycistronic readthrough transcripts from each fungus (Table 1). Among the *P*. *crispa* polycistronic gene pairs with homologous gene configurations in these species, PacBio long reads confirmed polycistronic transcripts associated with three gene pairs in *Trametes versicolor*, four gene pairs in *Phanerochaete chrysosporium* and one gene pair in *Gloeophyllum trabeum* (19, 19 and 21 total conserved gene pairs per species, respectively, Table E in S1 File). To provide support for the absence of polycistronic transcription in non-Agaricomycetes we analyzed a deep long-read transcriptome data set for the ascomycete *Neurospora crassa*[35] (*N*. *crassa*). The *N*. *crassa* PacBio transcriptome data set was 10 times higher depth than the non-*P*. *crispa* Agaricomycete data sets and comparable in depth to our *P*. *crispa* sequencing. Applying the same criterion and analysis as followed in *P*. *crispa*, we found no evidence of any PTUs in N. crassa. These results are consistent with the above hypothesis that genome-wide polycistronic transcription is likely to be prevalent among mushroom-forming Agaricomycetes but not inherent to our method nor is it a ubiquitous feature of fungal transcriptomes.

**Table 1:** Polycistronic transcripts identified in several fungi transcriptomes.

Organism	Order/Class	Poycistronic transcription
*Plicaturopsis crispa*	Agaricomycete/basidiomycete	229
*Phanerochaete chrysosporium*	Agaricomycete/basidiomycete	118
*Trametes versicolor*	Agaricomycete/basidiomycete	108
*Gloeophyllum trabeum*	Agaricomycete/basidiomycete	100
*Neurospora crassa*[35]	Sordariomycetes /ascomycete	0

## Discussion

Here we present a combined experimental and bioinformatics strategy (ToFU) that uses PacBio long reads for transcript isoform discovery. This strategy does not rely on reference genomes, and thereby enables the study of the transcriptome of any species. We showed that our strategy accurately reconstructs complex transcriptomes without relying on short-reads for error-correction or short-read assembly.

Lower mRNA isoform diversity has been observed in fungi compared to plants or animals. The low estimation is likely reflective of biased sampling from fungal lineages, such as the ascomycetes that have less complex gene structures and may have lower levels of isoform diversity[36]. The proficiency of ToFU was demonstrated on the transcriptome of the wood-degrading basidiomycete *P*. *crispa*. Our study shows that more than half of the genes in *P*. *crispa*, produce more than one transcript isoform, suggesting transcript isoform diversity in this phyla has likely been underestimated previously due to lack of deep full-length cDNA data. Similar to other non-fungal systems, genes producing the largest numbers of distinct isoforms are probable targets for regulation by NMD[22]. In this way alternative splicing may control production of functional proteins. Sequence optimization of GH and related enzymes may therefore be important in order to influence splicing and maximize production of transcript isoforms encoding functional enzymes in bioengineered systems.

A surprising finding is the discovery of long polycistronic transcripts spanning multiple independently transcribed loci that retain coding potential. Analysis of gene configuration conservation and long-read sequencing of multiple transcriptomes suggests that PTUs may be found widely in Agaricomycete fungi. PTU loci were recently inferred in yeast based on transcript end profiling[37]. This suggests that PTUs could be a conserved feature throughout the fungal kingdom. However, we show that PTUs are not present in the ascomycete N. crassa. PTUs found in P. crispa were enriched in tandem gene duplicates (hypergeometric test, p value < 1e-07). N. crassa has severely limited tandem gene duplication and repetitive element content due to highly efficient RIP[38]. Neither yeast nor the Agaricomycete fungi studied here are predicted to have stringent RIP[39]. Thus lack of PTUs in N. crassa may be a unique feature of that species determined by its lack of genome plasticity. Future long-read transcriptome studies in additional fungi that have efficient RIP will help resolve whether RIP may restrict PTUs or their expression. Interestingly, multi-ORF readthrough transcripts were associated with half of the bioinformatically detected secondary metabolite gene clusters identified by antiSMASH[40]. In the context of secondary metabolite gene clusters co-regulation and co-segregation can prevent the accumulation of toxic intermediates from these pathways[41]. Thus, PTUs may play an important role in achieving a particular ratio of enzymes produced from biosynthetic gene clusters, representing an advantageous mechanism to coordinate cellular responses.

A full understanding of the roles of PTUs requires further experimental characterization. For example, how are they translated? Are they post-transcriptionally cleaved and processed? Post-transcriptional processing in response to environmental conditions has been shown for specific cases in other systems to regulate protein expression[42]. Conducting heterologous expression and biochemical characterization of the products encoded by some of these PTUs is a crucial step to understand the function and role of PTUs in fungi. Manipulation and engineering of PTUs has the potential to positively impact the field of bioconversion.

## Methods

### Library preparation and cDNA sequencing

Total RNA was isolated from a monokaryotic culture of *Plicaturopsis crispa* grown on either a glucose-rich medium or microcrystalline cellulose. Total RNA was isolated from a monokaryotic cultures of *Phanerochaete chrysosporium*,*Trametes versicolor*, and *Gloeophyllum trabeum*. PolyA^+^ RNA was purified from total RNA via oligo-dT magnetic beads (Dynal). Four sequencing libraries (1–2 kb, 2–3 kb, 6kb, and no size-selection) were made for both P. crispa growth conditions and were prepared according to the PacBio isoform-sequencing protocol (http://www.smrtcommunity.com/servlet/servlet.FileDownload?file=00P7000000Pb1fkEAB). *Phanerochaete chrysosporium*, *Trametes versicolor*, and *Gloeophyllum trabeum* long-reads were generated from a size selected (>2 kb) cDNA library for each species. Single-molecule sequencing was performed on the PacBio RS II using P4-C2 chemistry, MagBead loading and 2 hour movie times. The previously described N. crassa data set[35] was generated on the PacBio RS II using P4-C2 chemistry from 60 SMRT cells.

### The ToFU pipeline

The pipeline consists of three stages: identifying full-length reads, isoform-level clustering, and final consensus polishing.

In the first stage, ToFU classifies all input raw reads into Circular Consensus Sequences (CCS) and non-CCS subreads by searching for the presence of sequencing adapters. Then ToFU determines a CCS or subread sequence to be full-length if both the 5’ and 3’ cDNA primers were present and there was a polyA tail signal preceding the 3’ primer.

In the second stage, ToFU uses an iterative isoform-clustering algorithm to cluster all the full-length reads derived from the same isoform. Briefly it first does clique-finding based on a similarity graph, then calls consensus using the Directed Acyclic Graph Consensus method and finally reassign sequences to different clusters based on their likelihood.

In the final stage, ToFU recruits the non-full-length reads and uses them to polish the consensus sequences produced during the second stage using the Quiver algorithm. Consensus sequences predicted to contain more than 10 errors are discarded.

### Merging the redundant PacBio transcripts into the ToFU transcript set

Due to the limitation of the cDNA library protocol, some cDNAs may not be full-length as they may lack the 5’-end. We collapsed transcripts that only differ in the 5’ start of their first exon but are otherwise identical in all subsequent exon structures keeping only the longest ones. The consequence of this step is that some transcripts with alternative transcription start sites are lost, but those with alternative splicing and alternatively polyadenylation will be preserved. This step can be avoided if the cDNA library protocol guarantees transcript sequences that preserve the 5’ start.

### Identification of polycistronic readthrough transcripts

We used Transdecoder for ORF prediction[43]. Transcripts with two or more non-overlapping ORFs ≥ 100 aa were further categorized based on reference annotations.

### RT-PCR and sequencing validation of the polycistronic RNAs

We selected 10 randomly selected polycistronic RNAs for experimental validation with the only criteria that specific primers of appropriate annealing temperature could be designed for the target. RT-PCR primers were designed so that the target region begins near the end of the first ORF and ends within the second ORF. RT-PCR products were pooled and sequenced by PacBio sequencing. 29,511 raw reads were aligned to the 10 reference transcripts using BLASR. Only high quality end-to-end alignments (19,706 reads) were further analyzed. Eight out of 10 RT-PCR products exactly matched the references and therefore validated the polycistronic RNAs. The remaining two were inclusive, as one (scaffold_9:1201061–1204786) did not yield any matching sequencing reads, while the other (scaffold_15:638864–642834) had a different 3’ end from the designated 3’ target site. These two may represent RT-PCR off-target cases. Further details are listed in Table D in S1 File.

### Poly-adenylation site (PAS) analysis

The poly-adenylation sites (PAS) of non-polycistronic and the second ORF of the polycistronic transcripts were identified by the polyA tail. The PAS of the first ORF of the polycistronic transcripts were identified with the aid of independent transcripts of the first ORF. The PAS motifs were predicted as previously described[30].

### Short-read transcript reconstruction

PolyA^+^ RNA was purified from the same total RNA samples as used for long-read sequencing. 100-bp paired end Illumina reads were generated on the HiSeq2000 according to the manufacturer’s instructions (Illumina). Short-reads were assembled using Rnnotator (v.3.0.0), Oases (v0.2.08), and Cufflinks (v.2.1.1). Rnnotator and Oases are both *de novo* transcript assemblers whereas Cufflinks is reference-based. In order to obtain optimal assembly results for Oases, we performed eight assemblies with Oases using different values of k-mer then used Vmatch (v2.2.0) to remove redundancy. The k-mer size ranged from 53 to 95 and the step size was 6. For Cufflinks, short-reads were first aligned to the reference genome with TopHat (v2.0.6) then the alignments were assembled into a parsimonious set of transcripts using Cufflinks. All three programs were run with default options with strand-specific information.

Assembled transcripts were mapped to the reference genome using GMAP (v2014-04-24) using parameters `—cross-species—allow-close-indels 0 –n 0`and filtered for ≥ 99% alignment coverage and ≥ 85% alignment identity; these parameters are the same as those applied to the PacBio consensus sequences. Finally, the same redundancy removal script used for collapsing PacBio consensus sequences was applied to create a non-redundant, high-quality transcript set for each assembly program.

### Conservation of homologous gene configurations of polycistronic-associated gene pairs in other sequenced fungi

For identification of cases of gene order conservation of polycistronic gene pairs in other fungi (Table E in S1 File), we searched for directly adjacent same-strand Blastp best hits in every fungal genome, publicly available at Mycocosm portal. From 288 possible adjacent ORFs in all transcripts, 78 P. crispa ORF pairs had conserved adjacent ORF pairs among the 47 publicly available fungal genomes. Conservation of strictly adjacent ORF pairs varied with a maximum of 25 conserved ORF pairs (32%).

## Supporting Information

S1 File Supplemental Text and Tables. PacBio sequencing statistics (Table A). Statistics for assembled transcripts from short reads (Table B). Comparison of assembled transcripts from short reads against PacBio transcripts (Table C). RT-PCR validation of polycistronic transcripts (Table D). The list of species that are used for searching conserved gene pairs (Table E).
(PDF)

S2 File Supplemental Figures. Most of the TOFU transcripts have longer UTRs than current annotation (Fig A). Genome-wide analysis of the transcription termination signals in *P*. *crispa* (Fig B).
(PDF)
