# Supplementary material for: Widespread Polycistronic Transcripts in Fungi Revealed by Single-Molecule mRNA Sequencing
Source: PLoS One. 2015 Jul 15;10(7):e0132628. doi: 10.1371/journal.pone.0132628 (PMC4503453; doi:10.1371/journal.pone.0132628)
Supplement: S1 File — PacBio sequencing statistics (Table A). Statistics for assembled transcripts from short reads (Table B). Comparison of assembled transcripts from short reads against PacBio transcripts (Table C). RT-PCR validation of polycistronic transcripts (Table D). The list of species that are used for searching conserved gene pairs (Table E). (PDF) [file pone.0132628.s001.pdf]

Supplementary Text

1. TOFU: a bioinformatics pipeline for PacBio transcriptome data

We developed a novel bioinformatics pipeline called TOFU to leverage both CCS (Circular Consensus Sequence) reads and non-CCS reads for transcript discovery. TOFU consists of three components: identifying full-length reads, isoform-level clustering, and final consensus polishing. We explain details in each step in the subsections below.

1.1 Full-length read identification and artifact removal

Given either CCS or subreads, we use the *phmmer* program from HMMER[1] to detect and remove the Clontech 5' / 3' primers (5' – AAGCAGTGGTATCAACGCAGAGTAC – 3'). A read is considered full-length if both primers are detected at the ends with a polyA tail signal of at least 12 consecutive 'A's preceding the 3' primer. Based on polyA tail and 3' primer orientation, primer-trimmed reads are reverse complemented to represent the sense strand. Because the Clontech protocol does not ensure the capture of the 5' cap, reads are considered 3'-complete but potentially 5'-partial; the 5' incompleteness is taken into account in later stages of transcript collapsing. To remove artificial concatemers that may have formed via ligation of primer-attached inserts, the same *phmmer* program is used to detect the presence of Clontech primers at least 100 bp away from either end of the sequence.

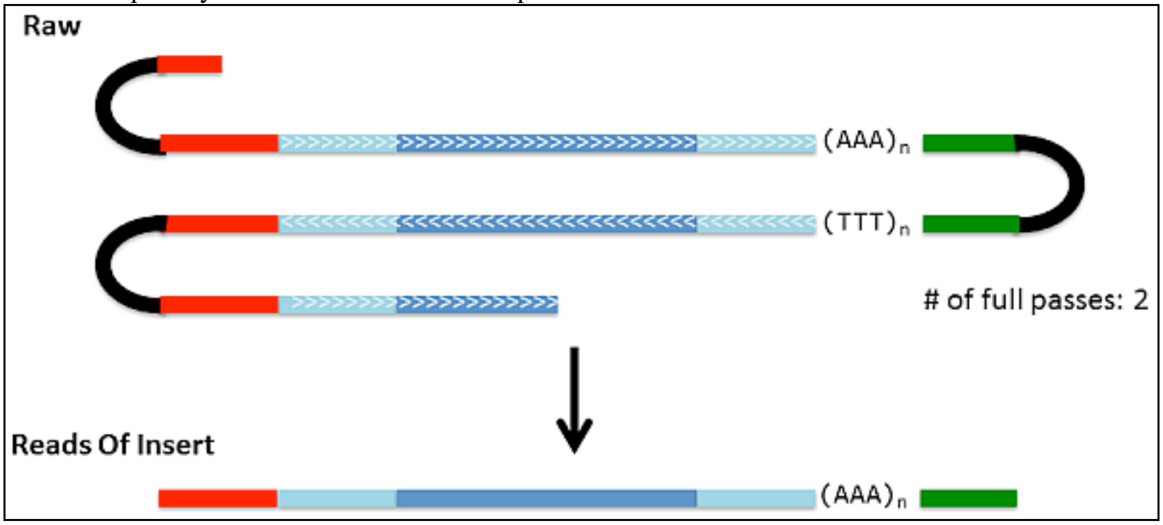

1.2. Iterative isoform clustering & consensus calling using Quiver

We develop an iterative isoform clustering algorithm called ICE (Iterative Clustering for Error Correction) that uses PacBio sequencing QVs for determining whether two reads come from the same isoform. ICE consists of several main modules: (1) clique-finding based on similarity graph; (2) fast consensus calling with no QV information using DAGCon; (3) reassignment of sequences to different clusters based on likelihood. The following flow chart shows the process.

In the initial phase of clustering, the input sequence, which are often only a portion of the entire dataset, are aligned against each other using BLASR[2] to construct a similarity graph where each node represents a read and each connecting edge indicates an "isoform hit". Since BLASR is designed to align through long stretches of gaps, a hit between two transcripts that share some number of exons may have an alignment. To distinguish alignments from the same isoform while accounting for sequencing errors, an alignment between two reads is considered an "isoform hit" (from the same isoform) only if the percentage of gaps that cannot be attributed to base errors within a window size *w* is below some threshold *T*.

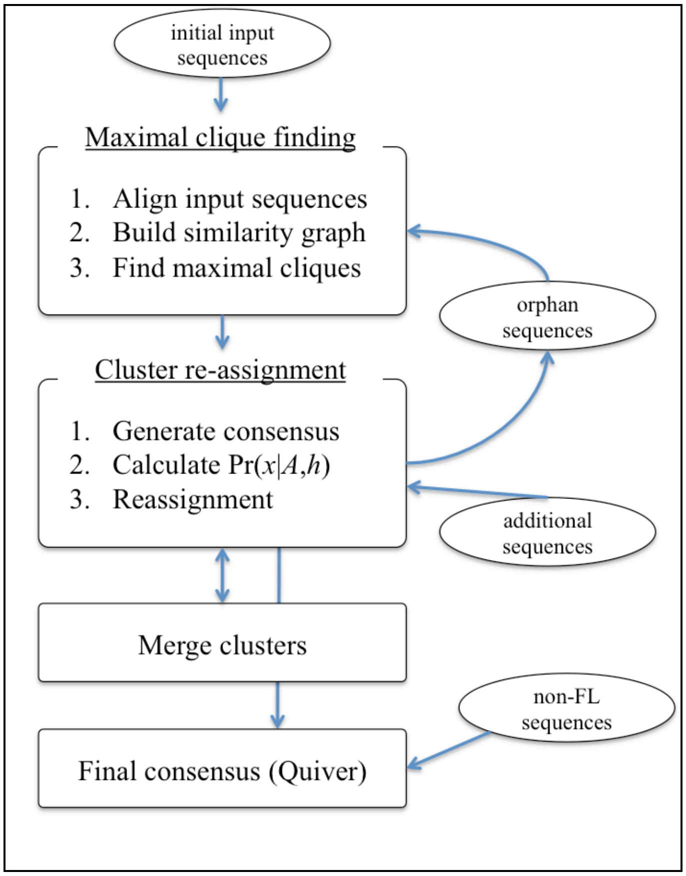

Formally, let the alignment string between two fully aligned sequences x and y be $A = a_1a_2...a_n$ where a_i is 'M' for a match, 'S' for a substitution, 'I' for insertion, and 'D' for deletion (hence A is just an unraveled cigar string). Let $p_x^{del}(i), p_x^{ins}(i), p_x^{sub}(i)$ denote the probability of each error type based on the raw QVs for sequence x. Construct an non-match vector $E = e_1e_2...e_n$ where $e_i = 0$ if one of the following is true:

- a_i = 'M'
- a_i = 'S' and ($p_x^{sub}(i_x) > c$ or $p_y^{sub}(i_y) > c$)
- a_i = 'I' and ($p_x^{ins}(i_x) > c$ or $p_y^{del}(i_y) > c$)
- a_i = 'D' and ($p_x^{del}(i_x) > c$ or $p_y^{ins}(i_y) > c$)

otherwise $e_i = 1$ which indicates a likely genuine non-match. Finally, we define x and y as being different isoforms if exists $i, j > 0$, where $j - i < w$, such that $\sum_{k=i..j} e_k \geq T * w$. In other words, we identify indel-rich regions in the alignment that are likely due to exon-level differences. We use a previously published linear time algorithm for identifying indel-rich regions[3] . A pair of aligned sequences x, y, that do not have an indel-rich region, is considered an "isoform hit". In this study, we set $c = 0.1$, $w = 20$, and $T = 0.5$.

With the similarity graph constructed using isoform hits, we look for perfect cliques in the graph. Ideally, all sequences from the same isoform would form a clique on its own with no other connecting edges. In practice, however, it is more likely that the sequences would form several cliques and may contain false positives (sequences from other isoforms). We address this by allowing "reassignment" of sequences to other clusters in a later step. For now, we run a maximal clique finding algorithm [4, 5] that non-deterministically finds maximal cliques in a graph, removes

the clique nodes from the graph, then repeat the process until the entire graph is partitioned into mutually exclusive cliques (clusters).

We call an initial consensus on all clusters using DAGCon, a directed acyclic graph based consensus calling algorithm originally developed for error correcting PacBio genomic sequences[6]. With the improved accuracy of the consensus sequences, we can better approximate the likelihood of sequences belonging to the same isoform. Here, we use a "reassignment" procedure similar to the Gibbs sampling method described for detecting HIV quasispecies[7]. Briefly, we calculate the posterior probability of a sequence *x* originating from an isoform *h*(*c*) for cluster *c* as:

$$\Pr(x|A,h(c)) = \prod_{i=1}^{n} (p_x^{mat}(i_x))^{(a_i='M')} \, (\tfrac{1}{3}p_x^{sub}(i_x))^{(a_i='S')} \, (\tfrac{1}{3}p_x^{ins}(i_x))^{(a_i='I')} \, (p_x^{del}(i_x))^{(a_i='D')}$$

Theoretically, we need to calculate $\Pr(x|A,h(c))$ for all sequences *x* and all cluster consensus $h(c) \in \mathbf{h}$. In practice, only pairs of (*x*, *h*(*c*)), for which there is an "isoform hit" are calculated. Here "isoform hit" uses the same linear time algorithm in the similarity graph construction; the only difference is *h*(*c*) is considered error-less.

At each "reassignment" step, for each sequence *x*, there are three possible moves:

- Case 1: If no isoform hit exists for *x*, it is put into an "orphan" bin
- Case 2a: If there exists another cluster *c'* such that $\Pr(x|A,h(c')) > \Pr(x|A,h(c))$, reassign *x* to *c'*.
- Case 2b: If *x* is in a singleton cluster and there exists another cluster *c'* such that *h*(*c'*) and *x* has an isoform hit, with some probability *p*, reassign *x* to *c'*.
- Case 3: If none of the above is true, *x* remains in *c*.

Case 2a deals with clusters that are big enough (>= 3 sequences) to generate consensus. In cases where DAGCon cannot generate consensus because there is only 1 or 2 sequences in the cluster (called "singleton clusters"), $\Pr(x|A,h(c))$ will always have the best probability and *x* will not have any possible moves. To allow the singletons to "escape", we reassign it to another cluster for which there is an isoform hit with some low probability (by default, *p*=0.3).

At the end of the reassignments, the "orphan" sequences go through the same similarity graph construction and maximal clique finding process to form new clusters. Any cluster that has membership changes must have run through DAGCon again for consensus calling, as well as $\Pr(x|A,h)$ recalculated.

Because our algorithm does not jointly optimize for a global objective function (such as $\Pr(\boldsymbol{h}|\boldsymbol{A},\boldsymbol{x})$, the total probability of observing the clusters given the input sequences and alignments) and our maximal clique finding is not guaranteed to put all isoform sequences in one cluster, a single isoform can end up being represented by multiple clusters. Thus, we add a phase of cluster merging, where the consensus sequences of two clusters are aligned against each other and if they are highly identical ($\geq 99\%$ similar) and are considered an isoform hit, then the two clusters are merged together. Note that, if two clusters were incorrectly merged, most commonly DAGCon will call consensus on one isoform but not the other, and as a result sequences belonging to the other isoform will be "orphaned" out in the next reassignment phase.

The iterative nature of the clustering process described so far makes adding new sequences very easy. New sequences can be introduced as follows: First, all new sequences are aligned against existing cluster consensus and assigned to the cluster with highest probability. For all sequences that did not have an isoform hit to an existing cluster, it is "orphaned" and follows the maximal clique finding procedure to be introduced into the dataset.

To summarize, the iterative process consists of maximal clique finding, consensus calling using DAGCon, cluster reassignment, and cluster merging. After a burn-in phase of reassignment and merging, the final set of DAGCon-generated consensus sequences are sent to the final stage of consensus calling using the more accurate and slower Quiver.

In this final stage of Quiver consensus calling, non-full-length reads, that were excluded from the iterative clustering process, is recruited to improve consensus accuracy. Non-full-length reads are aligned to all DAGCon-generated consensus sequences and filtered so that only "isoform hits" (using the same criterion as before but allowing for partial alignment) remain in the final alignment. Quiver uses the raw QVs from all aligned PacBio reads and outputs informative QVs along with the consensus sequence. Using the consensus QVs, we can filter out low quality consensus sequences that are often junk sequences and artifacts, though we also risk throwing out rare transcripts that have too little coverage.

Several speedups and parallelization are employed in the actual implementation of ICE. First, full-length reads are binned by size range (ex: 1-2k, 2-3k, 3-6k) since sequences from the same isoform must be within certain length differences even with indel errors. Partitioning the input sequences also serves to reduce the memory usage of each ICE process, which for efficiency maintains all QV information of "active" sequences (described below) in memory. Depending on the readlengths, 100k reads can take up 40-60GB of memory. Another speedup employed is to re-run DAGCon consensus calling only on clusters that are relatively small, where the removal of one or two sequences can affect the consensus sequence. In this study, we set the re-run cluster A, h does not to be re-calculated since it will remain the same. Finally, ICE maintains a set of "active" sequences that are sequences that are highly likely to be reassigned or orphaned because it is in a small cluster. A "freeze phase" is introduced after certain iterations of ICE, where any sequence that is in a cluster of size greater than the re-run size threshold and does not have an isoform hit to any other clusters is "inactivated" and forced to remain in its current cluster. QVs of inactive sequences are removed from memory. Consequently, clusters that contain inactive sequences, which must be of size greater than the re-run size threshold, does not ever have consensus re-run, and its core members can only increase.

1.3 Software availability

As of this writing, TOFU has been incorporated into the official SMRTAnalysis suite (versions 2.2 and above) by Pacific Biosciences under the protocol name RS_IsoSeq. The only difference between TOFU and RS_IsoSeq is that while TOFU uses a mixture of CCS reads and subreads, RS_IsoSeq uses the improved ReadsOfInsert protocol that generates a consensus read for each ZMW. The developemental version of TOFU is available publicly at http://github.com/PacificBiosciences/cDNA_primer.

2. Short read mapping to long read consensus and filtering by coverage

Strand-specific Illumina paired short reads are treated as paired and concordantly mapped to the PacBio consensus sequences using BowTie2 with '--very-fast –norc' and otherwise default parameters. Based on the short read coverage, PacBio consensus sequences are discarded if it: (1)

has zero short read coverage that is not at the end of the sequence; or (2) has a sudden drop in coverage that is greater than 100X fold and the smaller coverage is less than 10.

3. Identifying exon and splice junctions and removing redundancy
PacBio consensus sequences are mapped to the *P. crispa* contigs using GMAP (version 2014-04-24) with parameters '--allow-close-indels 0 --cross-species'. Alignments with less than 99% coverage are discarded. Exon boundaries and alternative junctions are defined based on the remaining alignments. Because the PacBio reads are considered 3' complete but possibly 5' partial, transcripts are merged if they share the same 3' exon and do not have any conflicting splice junctions. In the case of a single-exon transcript, all overlapping transcripts are merged.

4. ORF prediction and comparison with genome annotation
ORF prediction is done using TransDecoder[8] on the PacBio (TOFU) transcript consensus sequences. To find polycistronic candidates, we filter for any PacBio transcripts that satisfy the following: (1) has two or more non-overlapping ORF predictions; (2) does not have another PacBio consensus with a single ORF prediction that maps to the same loci and whose predicted ORF length is between 80%-120% of the total ORF length (in aa) from (1). We ignore any polycistronic candidates that have a similar PacBio consensus with single predicted ORF because most of them appear to be either incompletely or faulty spliced. The filtered polycistronic transcripts are then categorized as either reference-supported or non-reference-supported depending on whether each of the predicted ORFs overlaps an annotated reference gene.

5. Identification of full-length CCS and subread sequences
We ran a total of 77 SMRTCells on the PacBio RS II consisting of different size fractions: 20 with no size selection, 22 from the 1-2k size selection, 19 from the 2-3k size selection, and 16 from the > 3k size selection. The loading efficiency (P1) for the runs were from 30-55%, which is the recommended range. The RS_Filter protocol from SMRTPortal (version 2.0) was used to generate filtered CCS (Circular Consensus Sequence) and subread sequences. Out of a total of 4,920,305 sequencing ZMWs, 1,628,297 (33%) were CCS ZMWs. We defined a CCS or subread sequence to be full-length (FL) if both the 5' and 3' cDNA primers were present and there was a polyA tail signal preceding the 3' primer. Primers and polyA tails were trimmed from full-length sequences. Out of a total of 2,177,319 full-length CCS or non-CCS ZMWs, 4,748 were detected as artificial concatemers (0.2%) and were removed. The remaining trimmed, full-length sequences were further filtered for potential PCR chimeras by removing any sequence with at least 12 consecutive Ts in the beginning of the sequence. The remaining 2,548,103 sequences (from 2,143,039 ZMWs) were then used as input to the subsequent ICE clustering step.

6. Creating high-quality transcript consensus sequences
To speed up the clustering step, input sequences were divided into several bins (one for sequences shorter than 1kb, four for sequences between 1-2kb, two for sequences between 2-3kb, and one for sequences longer than 3kb) and ran through ICE independently on each bin. This resulted in many redundant transcripts consensus sequences that were merged in later steps. After obtaining the Quiver consensus sequence for each cluster, we estimated the number of expected errors based on the consensus QVs and discarded any consensus sequence that had more than 10 expected errors. While we risk throwing away rarer transcripts that have less coverage and thus worse consensus QVs, this ensured that the resulting consensus sequences were high quality. 40% of the clusters (176,903/443,242) passed this filter, which together consisted of 84% of the full-length input sequences. Most of the discarded clusters consisted of only one subread sequence, suggesting that these were likely low-quality sequences.

The high-quality consensus sequences were mapped to the *P. crispa* genome scaffolds using GMAP (version 2013-07-20) and removed for any sequence that did not map to the genome with at least 99% coverage; 12,085/179,603, or 6.8%, were removed.

7. Further filtering of PacBio consensus sequences based on short read evidence
Paired-end Illumina reads were mapped to the PacBio consensus sequences using BowTie2. Most of the short reads mapped to at least one PacBio consensus sequence. We found that most PCR chimeras that have formed during the full-length cDNA library construction in the PacBio reads were successfully filtered by the detection for polyT stretches in the sequence filtering steps. To exclude remaining PCR chimeras, we discarded any consensus sequence that did not have sufficient Illumina short read coverage throughout the sequence. We removed 17,335 out of 164,818 consensus sequences at this step. The remaining 147,483 consensus sequences then constitutes the redundant, high-quality transcript sequences that are supported by three independent sources: PacBio raw read support, Illumina short read support, and good alignment to the reference genome.

8. Collapsing redundant PacBio transcripts
Because the PacBio reads were considered 3' complete but possibly 5' partial, transcripts were merged if they shared the same 3' exon and did not have any conflicting splice junctions. After merging redundant transcripts, we obtained 22,956 non-redundant transcript sequences in 9,073 isoform clusters.

9. Categorizing polycistronic readthrough transcripts
We screened for PacBio transcripts that had two or more non-overlapping ORF predictions that were not covered by another transcript that had a single, long ORF prediction. We then collapsed the readthrough transcripts by their mapped genomic locations and found 508 distinct loci to be polycistronic, among which 314 have support from genome-based gene predictions. The polycistronic transcripts were distributed across the genomic scaffolds and ranged from 828 to 5080 bp with an average length of 2330 bp. The majority of the candidates were bi-cistronic (471/508, or 93%), with the mean ORF lengths for the first and second ORFs being 256 aa and 277 aa .The mean distance between the two ORFs was 364 bp.

Supplementary Tables

Table A. PacBio Sequencing

**RS II sequencing statistics statistics.**	
Number of SMRTcells	77
Number of sequencing ZMWs	4,920,305
Number of full-length ZMWs	2,177,319
**Quality filtering**	
Removed: artificial concatemers	4,748 (0.2%)
Removed: suspicious polyT	29,532 (1%)
Input to consensus clustering (ICE): 2,548,103 sequences (2,143,039 ZMWs)	
**Post-ICE consensus sequencing filtering**	
Number of clusters	443,342
Number of high-quality clusters	176,903 (40%)
Removed: low alignment to genome	12,085 (6.8%)
Removed: low short read support	17,335 (10.5%)
Number of high-quality, redundant clusters with support from PacBio, Illumina short reads, and genome scaffold: 147, 483	
**Collapsing redundant transcripts**	
Number of non-redundant transcripts	22,956
Number of isoform clusters	9,073

Table B. Statistics for assembled transcripts from short reads.

**Program**	**Number of assembled transcripts**	**Well-mapped transcripts**	**Number of Non-redundant transcripts**	**Length of non-redundant transcripts (nt)**		
				Min	Max	Median
Rnnotator	29,754	27,549	24,637	43	15,374	614
Oases	112,669	80,761	68,693	100	19,699	1,481
Cufflinks	10,211	10,184	10,051	96	20,688	1,960

Table C. Comparison of assembled transcripts from short reads against PacBio transcripts.

**Program**	**Number of non-redundant transcripts**	**Match against PacBio***					
		**Exact**	**Extended**	**Subset**	**Concordant**	**Alternative**	**Nomatch**
Rnnotator	24,637	3975 (16%)	2488 (10%)	5770 (23%)	824 (3%)	5171 (21%)	6409 (26%)
Oases	68,693	5212 (17%)	20419 (33%)	14351 (6%)	4581 (3%)	11081 (22%)	13049 (19%)
Cufflinks	10,051	1684 (8%)	3308 (30%)	590 (21%)	294 (7%)	2220 (16%)	1955 (19%)

* Each assembled transcript was matched against PacBio transcripts and categorized based on the number and exact position of donor-acceptor sites, regardless of the start and end position of the first and last exon. An exact match indicates that the exon junctions are the same, whereas extended, subset, and concordant indicates exon junction agreement where there is overlap but there are additional junctions not covered by either the assembled transcript or PacBio. An alternative match means disagreement in exon junctions but the mapped loci overlaps. Finally, a nomatch indicates no PacBio is observed at the loci.

Table D. RT-PCR validation of polycistronic transcripts. Primers were designed to cover more than one of the predicted ORF regions to prevent mis-validation by sequencing of non-polycistronic transcripts that contain only one of the ORFs. Eight out of ten of the RT-PCR products and subsequent sequencing confirmed the presence of the polycistronic transcripts.

Transcript ID	Length (nt)	Locus	Primer target region	Observed read region	Number of Full-Match Reads		
					5' primer detected	3' primer detected	5' & 3' detected
i1_c21309	1918	scaffold_13:451943-454078	635 - 1832	635 - 1832	91	123	71
i4_c15846	2155	scaffold_4:1364493-1367235	393 - 1784	393 - 1784	77	104	45
i4_c2393	2949	scaffold_16:256387-259704	437 - 2428	437 - 2428	432	405	312
i4_c71086	2728	scaffold_9:1201061-1204786	30 - 2029	30 - NA	6989	0	0
i5_c14860	2457	scaffold_5:113696-116791	110 - 1606	110 - 1606	930	871	747
i6_c15213	3333	scaffold_3:1270271-1274332	1006 - 2505	1006 - 2505	113	137	93
i6_c18769	3332	scaffold_8:1460341-1464471	398 - 2593	398 - 2593	652	607	495
i6_c19101	4168	scaffold_14:668239-673734	1622 - 3821	1622 - 3821	206	208	157
i6_c36760	3101	scaffold_2:123125-126961	125 - 2426	125 - 2426	64	49	31
i6_c38512	3506	scaffold_15:638864-642834	382 - 3390	382 - 1860	1290	1233	1071

Table E. The list of species that are used for searching conserved gene pairs.

JGI mycocosym ID	conserved gene pair configurations	taxonomic relation to *P. crispa*
Ompol1	10	Agaricales
PleosPC9_1	21	Agaricales
Agabi_varbisH97_3	19	Agaricales
Schco_LoeD_1	12	Agaricales
Schco3	12	Agaricales
Schco_TatD_1	10	Agaricales
Lacbi2	23	Agaricales
Volvo1	7	Agaricales

Agabi_varbur_1	18	Agaricales
PleosPC15_2	16	Agaricales
Agabi_varbisH97_2	17	Agaricales
Galma1	19	Agaricales
Armme1	7	Agaricales
Punst1	15	Agaricomycetes
Conpu1	20	Agaricomycetes
Phchr2	19	Agaricomycetes
Aurde3_1	3	Agaricomycetes
SerlaS7_3_2	22	Agaricomycetes
Botbo1	4	Agaricomycetes
Dicsq1	13	Agaricomycetes
Wolco1	14	Agaricomycetes
Bjead1_1	18	Agaricomycetes
PosplRSB12_1	14	Agaricomycetes
Fompi3	17	Agaricomycetes
Jaaar1	25	Agaricomycetes
Glotr1_1	21	Agaricomycetes
Cersu1	17	Agaricomycetes
SerlaS7_9_2	24	Agaricomycetes
Stehi1	16	Agaricomycetes
Pirin1	1	Agaricomycetes
Phlbr1	15	Agaricomycetes
Phaca1	17	Agaricomycetes
Trave1	19	Agaricomycetes
Hetan2	22	Agaricomycetes
Serla_varsha1	21	Agaricomycetes
Fomme1	13	Agaricomycetes
Gansp1	15	Agaricomycetes
Dacsp1	3	Agaricomycotina
Treme1	1	Agaricomycotina
Psehu1	1	Basidiomycota
Mellp1	1	Basidiomycota
Tilan2	1	Basidiomycota
Malgl1	2	Basidiomycota
Hisca1	1	Ascomycota
Clagr3	1	Ascomycota
Hyspu1	1	Ascomycota
Talma1_2	1	Ascomycota

References

1. Eddy, S.R. Accelerated Profile HMM Searches. *PLoS Comput Biol* **7**, e1002195 (2011).
2. Chaisson, M.J. & Tesler, G. Mapping single molecule sequencing reads using basic local alignment with successive refinement (BLASR): application and theory. *BMC Bioinformatics* **13** (2012).
3. Tseng, H.H. & Tompa, M. Algorithms for locating extremely conserved elements in multiple sequence alignments. *BMC Bioinformatics* **10**, 432 (2009).
4. Abello, J., P. M. Pardalos, and M. G.C Resende On Maximum Clique Problems in Very Large Graphs. *AT&T Labs Reserrch Technical Report* **TR98** (1998).
5. Tseng, H.-H. Discovery and Applications of Bacterial Noncoding RNAs. *PhD thesis, Department of Computer Science & Engineering: University of Washington* (2012).
6. Chin, C.S. et al. Nonhybrid, finished microbial genome assemblies from long-read SMRT sequencing data. *Nat Methods* **10**, 563-569 (2013).
7. Zagordi, O., Geyrhofer, L., Roth, V. & Beerenwinkel, N. Deep sequencing of a genetically heterogeneous sample: local haplotype reconstruction and read error correction. *J Comput Biol* **17**, 417-428 (2010).
8. Haas, B.J. et al. De novo transcript sequence reconstruction from RNA-seq using the Trinity platform for reference generation and analysis. *Nat Protoc* **8**, 1494-1512 (2013).
