# Supplementary material for: Widespread Polycistronic Transcripts in Fungi Revealed by Single-Molecule mRNA Sequencing
Source: PLoS One. 2015 Jul 15;10(7):e0132628. doi: 10.1371/journal.pone.0132628 (PMC4503453; doi:10.1371/journal.pone.0132628)
Supplement: S2 File — Most of the TOFU transcripts have longer UTRs than current annotation (Fig A). Genome-wide analysis of the transcription termination signals in P. crispa (Fig B). (PDF) [file pone.0132628.s002.pdf]

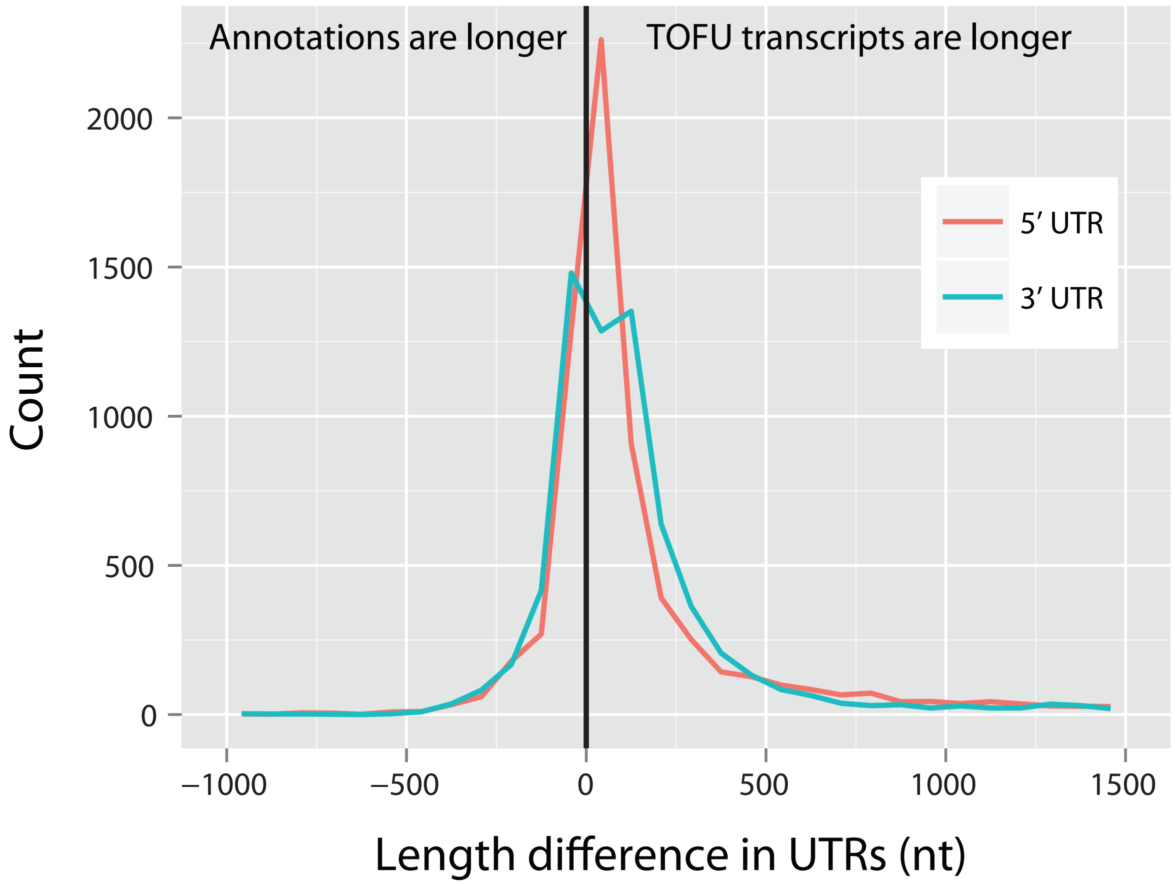


Figure A. Most of the TOFU transcripts have longer UTRs than current annotation.

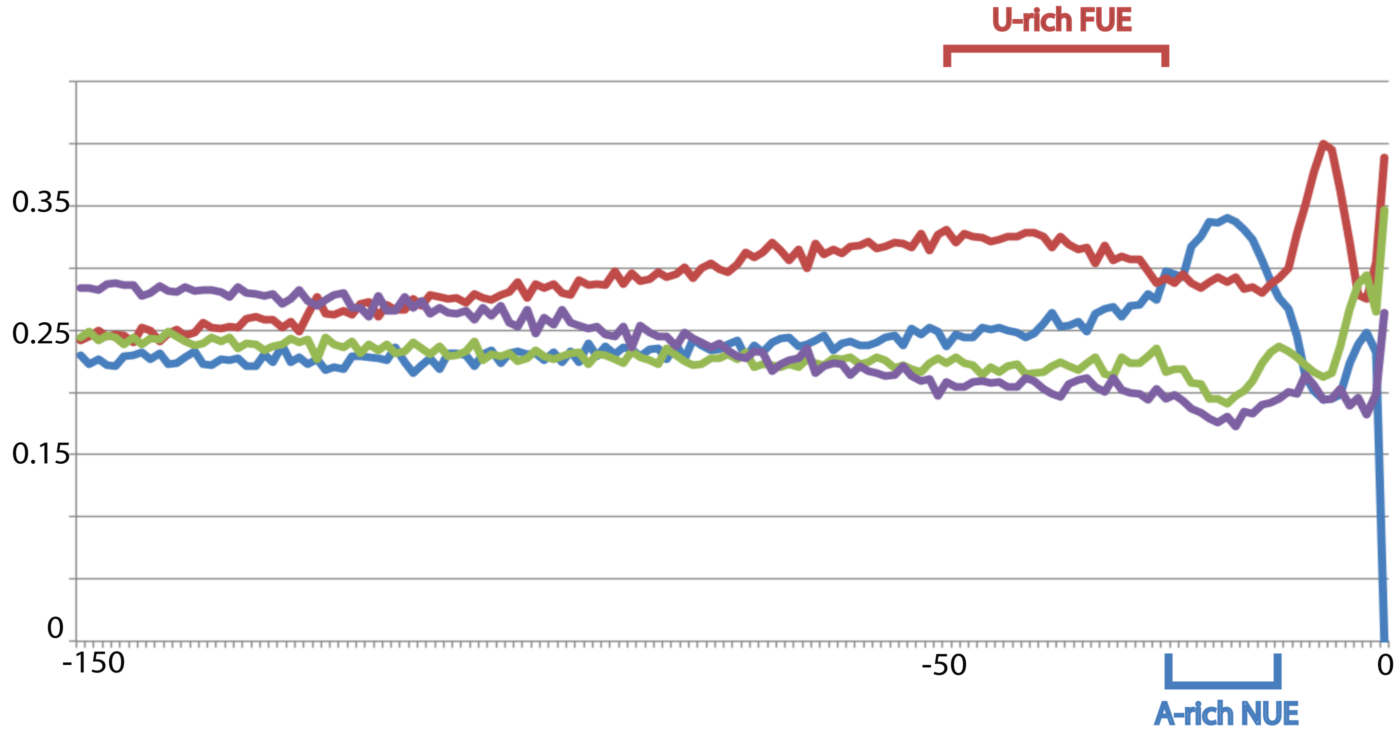


Figure B. Genome-wide analysis of the transcription termination signals in P. crispa. Average nucleotide composition was plotted for all non-polycistronic ToFU transcripts upstream of the poly-adenylation site. A-rich NUE and U-rich FUE elements are indicated. Green represents C-content and purple represent G-content, respectively. X-axis indicates the distance to polyA site.
